# Morphometric data for five freshwater turtles in south, central, and west Texas

**DOI:** 10.1016/j.dib.2020.105356

**Published:** 2020-02-29

**Authors:** Donald J. Brown, Ivana Mali, Melissa C. Jones, Michael R.J. Forstner

**Affiliations:** a School of Natural Resources, West Virginia University, Morgantown, WV, 26506, USA; b Northern Research Station, U.S.D.A. Forest Service, Parsons, WV, 26287, USA; c Department of Biology, Eastern New Mexico University, Portales, NM, 88130, USA; d Natural Resources Program, Texas Parks and Wildlife Department, Brownsville, TX, 78521, USA; e Department of Biology, Texas State University, San Marcos, TX, 78666, USA

**Keywords:** Carapace, Plastron, Weight, *Apalone spinifera emoryi*, *Chelydra serpentina*, *Kinosternon flavescens*, *Trachemys gaigeae*, *Trachemys scriptaelegans*

## Abstract

From 2008 to 2013, we sampled freshwater turtle populations at 66 sites in south, central, and west Texas, USA. Sampling sites included ponds, lakes, resacas (oxbow lakes), canals, and rivers. We sampled turtle populations using baited hoop nets (66 sites) and basking traps (3 sites), and captured turtles by hand opportunistically in terrestrial habitat. We measured carapace length and width, plastron length and width, body depth, and weight of captured turtles. Excluding recaptures, we measured 356 *Apalone spinifera emoryi* (Texas Spiny Softshell), 24 *Chelydra serpentina* (Snapping Turtle), 20 *Kinosternon flavescens* (Yellow Mud Turtle), 47 *Trachemys gaigeae* (Big Bend Slider), and 1070 *Trachemys scripta elegans* (Red-eared Slider). Carapace length of *Apalone spinifera emoryi* ranged from 85 to 426 mm (mean = 182 mm). Carapace length of *Chelydra serpentina* ranged from 74 to 320 mm (mean = 233 mm). Carapace length of *Kinosternon flavescens* ranged from 64 to 147 mm (mean = 114 mm). Carapace length of *Trachemys gaigeae* ranged from 54 to 203 mm (mean = 141 mm). Carapace length of *Trachemys scripta elegans* ranged from 30 to 328 mm (mean = 171 mm). These data are useful for assessing spatial and temporal variation in size and body condition of freshwater turtles.

**Table:** Specifications Table

Subject	Ecology, Evolution, Behavior and Systematics
Specific subject area	Herpetology, Morphometrics, Wildlife Biology, Zoology
Type of data	Figure, Table, Supplementary Spreadsheet
How data were acquired	Hoop-net traps, basking traps, and hand captures
Data format	Raw
Parameters for data collection	Ponds, lakes, resacas (oxbow lakes), canals, rivers, and terrestrial habitat
Description of data collection	Turtle data include species, sex, midline carapace length, midline carapace width, midline plastron length, pectoral scute plastron width, maximum body depth, and weight
Data source location	Texas, USA
Data accessibility	Repository name: Mendeley Data Data identification number: 10.17632/8n4x87fctp.3 Direct URL to data: https://data.mendeley.com/datasets/8n4x87fctp/3
Related research article

**Table:** 

**Value of the Data** • The data are useful for assessing spatial and temporal variation in size and body condition of freshwater turtles. • The data are beneficial to herpetologists and other wildlife researchers studying size variation. • The data can be used to improve our understanding of geographic or habitat-based variation in turtle size distributions and body condition, and serves as baseline data to study morphometric responses of turtles to climate change.

## 1 Data description

**Fig. 1:**
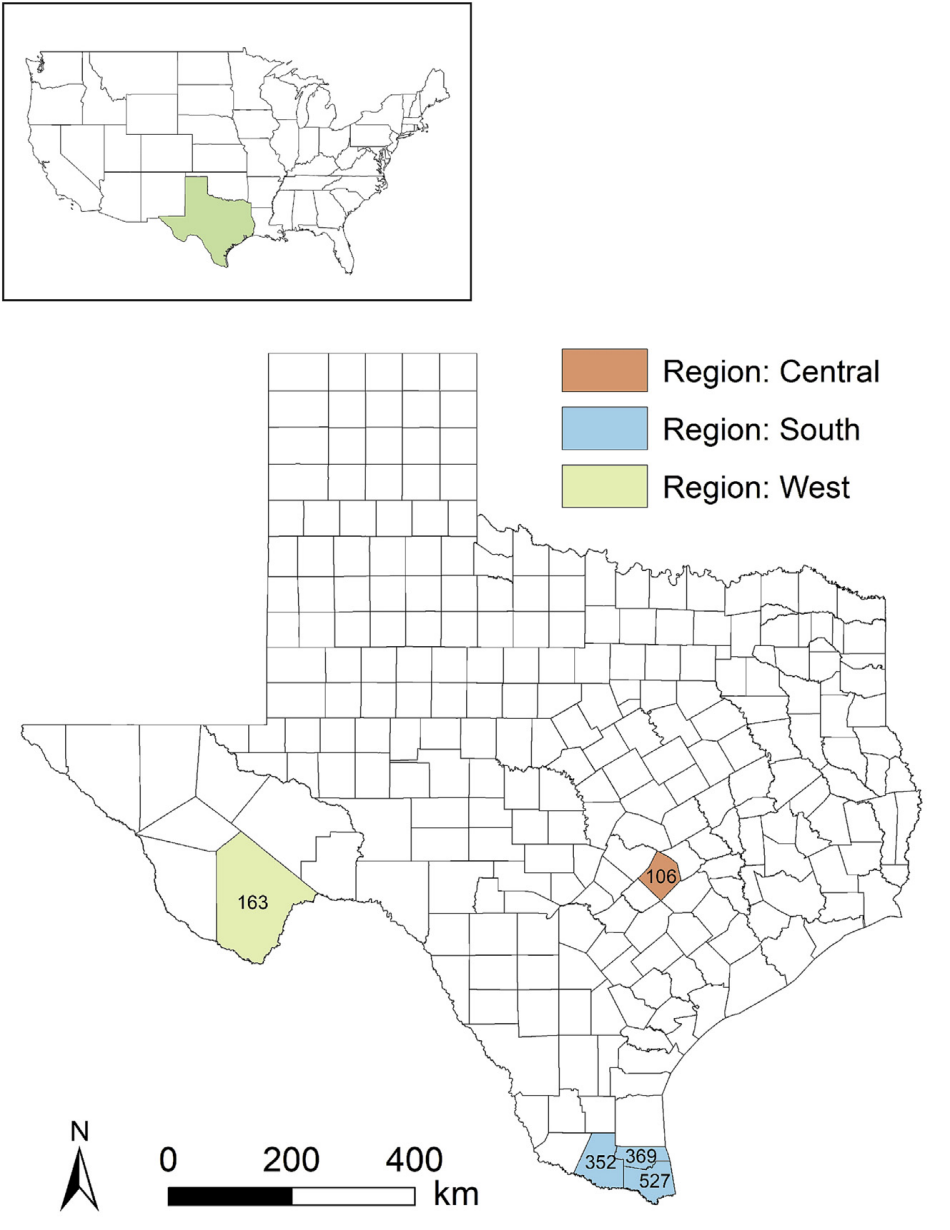
Regions and counties in Texas where we measured freshwater turtles. Numbers represent the total sample size per county.

The dataset [1] includes 1517 unique individual measurements for five freshwater turtle species in Texas, including 356 *Apalone spinifera emoryi* (Texas Spiny Softshell), 24 *Chelydra serpentina* (Snapping Turtle), 20 *Kinosternon flavescens* (Yellow Mud Turtle), 47 *Trachemys gaigeae* (Big Bend Slider), and 1070 *Trachemys scripta elegans* (Red-eared Slider). We performed measurements in south, central, and west Texas (Fig. 1). The file includes midline carapace length, midline carapace width, midline plastron length, pectoral scute plastron width, maximum body depth, weight, sex, date, location (region, county, site name, and coordinates), type of water body, capture method, and individual shell notch number. We measured turtles to the nearest 1 mm. We weighed turtles to the nearest 1 g, 5 g, 10 g, and 50 g for turtles weighing ≤10 g, ≤600 g, ≤2500 g, and >2500 g, respectively. Descriptive statistics for the morphometric data are shown in Table 1. A data key with additional information and scute notch diagram is included in the dataset [1] (Specifications Table).

**Table 1:** Descriptive statistics for morphometric data collected from 1517 unique individuals representing five freshwater turtle species in south, central, and west Texas. Measurements include midline carapace length (mm [CL]), midline carapace width (mm [CW]), midline plastron length (mm [PL]), pectoral scute plastron width (mm [PW]), maximum body depth (mm [BD]), and weight (g). Data include the mean, minimum, and maximum value recorded.

Measurement	*Apalone spinifera emoryi*	*Chelydra serpentina*	*Kinosternon flavescens*	*Trachemys gaigeae*	*Trachemys scripta elegans*
CL	182 (85–426)	233 (74–320)	114 (64–147)	141 (54–203)	171 (30–328)
CW	145 (70–330)	207 (60–274)	82 (51–103)	108 (50–152)	129 (31–220)
PL	135 (17–307)	168 (47–250)	101 (56–125)	127 (50–188)	157 (29–267)
PW	104 (41–235)	161 (45–224)	68 (41–87)	83 (2–125)	102 (21–175)
BD	47 (20–129)	104 (30–144)	46 (24–60)	50 (24–84)	70 (13–122)
Weight	731 (50–7400)	2013 (100–7600)	288 (50–540)	391 (25–1260)	913 (6–3100)

## 2 Experimental design, materials, and methods

From 2008 to 2013, we sampled freshwater turtle populations at 66 sites in south, central, and west Texas, USA, including ponds, lakes, resacas (oxbow lakes), canals, and rivers. We primarily captured turtles using 76.2 cm diameter single-opening, single-throated, widemouth hoop-nets with a 2.54 cm mesh size and four hoops per net (Memphis Net & Twine Co., Memphis, Tennessee, USA). Traps were kept taut using wooden posts connected to the first and last hoop. Two stretcher posts were used for each trap, located lateral to the mouth opening. We baited all traps, typically with sardines, in non-consumable containers containing holes for scent escape. We re-baited traps every 1 or 2 days. We placed flotation devices between the two middle hoops to prevent drowning and keep traps parallel with the water's surface. The number of traps and number of days spent trapping varied among sites and years, depending on study objectives [[2], [3], [4], [5], [6], [7], [8], [9]]. We obtained additional captures using basking traps at three of the sites, and by hand capture in terrestrial habitats (typically along roads).

For captured turtles, we measured midline carapace length [10], midline carapace width, midline plastron length [10], pectoral scute plastron width, and maximum body depth using tree calipers (Haglof, Madison, Mississippi, USA). We weighed turtles using spring scales (Pesola, Baar, Switzerland). We individually marked turtles by notching the carapace using a rotary tool (Dremel, Racine, Wisconsin). We determined sex using secondary sexual characteristics [11]. Habitat-related information for trapping sites can be found in our published articles [[2], [3], [4], [5], [6], [7], [8], [9]].

## References

[1] Brown D.J. (2019). https://data.mendeley.com/datasets/8n4x87fctp/3.

[2] Brown D.J., Schultz A.D., Dixon J.R., Dickerson B.E., Forstner M.R.J. (2012). Decline of red-eared sliders (*Trachemys scripta elegans*) and Texas spiny softshells (*Apalone spinifera emoryi*) in the Lower Rio Grande Valley of Texas. Chelonian Conserv. Biol..

[3] Brown D.J., DeVolld B., Forstner M.R.J. (2011). Escapes from hoop nets by red-eared sliders (*Trachemys scripta*). SW. Nat..

[4] Brown D.J., Mali I., Forstner M.R.J. (2011). No difference in short-term temporal distribution of trapping effort on hoop net capture efficiency for freshwater turtles. SE. Nat..

[5] Brown D.J., Farallo V.R., Dixon J.R., Baccus J.T., Simpson T.R., Forstner M.R.J. (2011). Freshwater turtle conservation in Texas: harvest effects and efficacy of the current management regime. J. Wildl. Manag..

[6] Mali I., Dickerson B.E., Brown D.J., Dixon J.R., Forstner M.R.J. (2013). Road density not a major driver of red-eared slider (*Trachemys scripta elegans*) population demographics in the Lower Rio Grande Valley of Texas. Herpetol. Conserv. Biol..

[7] Mali I., Brown D.J., Ferrato J.R., Forstner M.R.J. (2014). Sampling freshwater turtle populations using hoop nets: testing potential biases. Wildl. Soc. Bull..

[8] Mali I., Brown D.J., Jones M.C., Forstner M.R.J. (2012). Switching bait as a method to improve freshwater turtle capture and recapture success with hoop net traps. SE. Nat..

[9] Mali I., Brown D.J., Jones M.C., Forstner M.R.J. (2013). Hoop net escapes and influence of traps containing turtles on Texas spiny softshell (*Apalone spinifera emoryi*) captures. Herpetol. Rev..

[10] Iverson, J.B., Lewis, E.L., How to measure a turtle, Herpetol. Rev. 49(3) 453–460.

[11] Ernst C.H., Lovich J.E. (2009). Turtles of the United States and Canada.

